# Potential Issues in Mandating a Disclosure of Institutional Investigation in Retraction Notices

**DOI:** 10.1007/s11948-024-00468-2

**Published:** 2024-01-23

**Authors:** Bor Luen Tang

**Affiliations:** Department of Biochemistry, Yong Loo Lin School of Medicine, National University of Singapore, Singapore, Singapore

**Keywords:** Disclosure, Retraction, Retraction notice, Research misconduct

## Abstract

A retraction notice is a formal announcement for the removal of a paper from the literature, which is a weighty matter. Xu et al. (Science and Engineering Ethics, 29(4), 25 2023) reported that 73.7% of retraction notices indexed by the Web of Science (1927–2019) provided no information about institutional investigations that may have led to the retractions, and recommended that Committee on Publication Ethics (COPE) retraction guidelines should make it *mandatory* to disclose institutional investigations leading to retractions in such notices. While this recommendation would add to the transparency of the retraction process, a blanket mandate as such could be potentially problematic. For research misconduct (RM)-positive cases, a mandatory investigative disclosure may be abused by some to deflect responsibility. More importantly, a mandatory disclosure could harm authors and institutions in RM-negative cases (i.e. those stemming from honest errors with no misconduct). I illustrate with case vignettes the potential epistemic injustice and confusion that a mandate for investigation disclosure in retraction notices could incur, and suggest a more nuanced approach to its implementation.

## Introduction

When errors and irregularities are found in a scientific paper such that the data/results or conclusions of the paper are either no longer valid or could not be trusted, the last course of action would be to retract the paper. Such a retraction signifies a formal removal of the paper from the literature, and is typically announced via a retraction notice in the journal. A retraction may either be author-initiated (and mutually agreed between authors and editor)^1^ or may be unilaterally editorial^2^. The wordings of retraction notices of the former type could be one that is agreed upon by the authors and the editor, while those of the latter type are usually drafted by the editorial office, perhaps with a note as to whether each of the co-authors agree or disagree with the retraction. Retraction notices vary in length and details, but the reason for retraction would usually be briefly described.

The nature and characteristics of retraction notices have been the topics examined in several recent papers (Moylan & Kowalczuk, 2016; Vuong, 2020; Hesselmann & Reinhart, 2021; Teixeira da Silva & Vuong, 2022; Xu et al., 2023; Xu & Hu, 2023). A general concern voiced is that such notices tend to be brief and are insufficiently informative or transparent. For example, Vuong (2020) has noted from retraction notices of 2,046 retracted papers that 53% of these do not specify who initiated the retraction, and that nearly 10% of these do not contain information related to reasons for retractions. Notices may also be obscured on the occurrence of research misconduct (RM) (Hesselmann & Reinhart, 2021). RM, as defined by the US Office of Research Integrity, entails “fabrication, falsification, or plagiarism in proposing, performing, or reviewing research, or in reporting research results” (https://ori.hhs.gov/definition-research-misconduct). These definitions are widely adopted by the research community. However, other misdemeanours, transgressions or offences that occur in a research setting such as mistreatment of animal/human research subjects (https://www.councilscienceeditors.org/3-1-description-of-research-misconduct) and harassment of co-workers (Marín-Spiotta, 2018) are also often considered as RM. Of course, RM is only one reason whereby a paper could or should be retracted. Other than honest errors noted retrospectively, retraction could also occur for studies due to revelation of noncompliance with regulatory requirements (such as proper documentation of institutional ethics approval), duplicate publications or a compromised peer review process.

Writing in the pages of *Science and Engineering Ethics*, Xu and colleagues reported that 73.7% of retraction notices indexed by the Web of Science (1927–2019) provided no information about institutional investigations that may have led to the retractions (Xu et al., 2023). Xu et al. noted that their findings “… *points to a substantial lack of transparency about the decision-making process of retraction*”. The authors have thus recommended that the Committee on Publication Ethics (COPE) retraction guidelines (COPE council, 2019) should make it *mandatory* to disclose institutional investigations leading to retractions in such notices.

There are situations that might preclude the inclusion of a statement on institutional investigation, such as legal constraints posed by authors (Elia et al., 2014). Only some journals and editors go beyond examining the submitted materials, and journals would usually request for more detailed investigations, particularly when RM is suspected, by the authors’ hosting institutions. Institutional investigations tend to take time, and may be conducted or concluded at a much longer timeframe than that required for the retraction of a clearly problematic paper. While I agree in principle with Xu et al.’s recommendation, I shall explore whether such a mandatory disclosure of institutional investigation, or at least its blanket implementation, might be problematic or even undesirable. I suggest that the most important information in a retraction notice should be the proximal cause for the retraction, namely, a sufficiently detail explanation on why data/results/conclusions are no longer valid or trustworthy, in part or in whole, due to readily verifiable mistakes and errors (be it intended or otherwise). Furthermore, a paper should be retracted because there are fundamental faults with its content and not because of the outcome of institutional investigations, nor should a lack of such investigations preclude or delay its retraction. Importantly, not mandating the inclusion of such an investigation disclosure at the point of retraction would avoid incidences of epistemic injustice and confusion that might unnecessarily damage the reputation of the author(s) and institution(s), or undermine trust in science. I shall illustrate these issues below with some case vignettes.

## Cases and Scenarios

In all likelihood, retractions involving some form of RM would have gone through some formal investigations.^3^ However, this may or may not be stated or outlined in the retraction notice. Likewise, retraction not due to misconduct could also have been investigated, and again this investigation may or may not be mentioned in the retraction notice. For the RM-positive cases, a formal statement on the investigation and adjudication would add credence to the retraction and punishes the perpetrator. I do not see a problem with this, except to note that in some instances such investigation disclosures might place emphasis or focus only on the perpetrator(s), and inadvertently exonerate other parties (such as principal investigators and senior co-authors) that are also supposed to take responsibility^4^.

On the other hand, for the RM-negative cases, I do see a potential for the occurrence of epistemic injustice and confusion, which may harm the authors and would be undesirable for all parties involved. Such epistemic injustice and confusion could arise from the inability of an author to freely communicate with others on the details of a case when an investigation is underway. It could also arise among the public who are not factually informed of the details of the case, but are instead influenced by rumours and hearsays. I shall illustrate these possibilities with the following three fictitious^5^ cases.

In case 1, whistleblowers have highlighted instances of data and image irregularities of a paper in online forums and in written complaints to the journal. The journal reached out to the senior corresponding author A and all co-authors. Failing to receive a satisfactory explanation from A and his co-authors, the journal alerted A’s hosting institution, which then conducted a formal investigation. The institutional panel concluded that one of the junior co-authors has falsified and fabricated data in the paper as well as in another paper, and adjudicated the prompt retraction of these papers by A. A wrote to the journal to request for a retraction, expressing regret that he did not catch the issues earlier. The retraction notice accordingly included a brief disclosure of the institution’s investigation results, with the perpetrator named. In a public statement to the media, A has expressed regret for his misplaced trust in his underling, apologized for the inconvenience the case has caused, and indicated relief that the matter has been resolved.

In case 2, colleagues in the field had voiced their concerns with researcher B with regards to data and analyses in his recent paper. B re-analysed all his data, and realized that an error in his code has significantly exaggerated the final numbers, which when corrected pointed to a less pronounced effect than that originally reported in the paper. B contacted the editor, explained the mistake and requested to publish a correction. However, the editor refused the request for correction and instead asked for the “standard procedure” be followed. He requested, among other information, an official report of investigation from B’s host institution so that he could decide whether the paper should be retracted. B was caught off guard by the request. What he thought of as a scientific issue had been quickly and inadvertently escalated into a matter of RM.

In case 3, researcher C has published a paper which made a bold and novel claim based on limited data, and against apparently contrasting findings by others. A whistleblower has written to both the journal and researcher C’s institution with notification of perceived errors and insinuations of misconduct in the paper. The institution conducted an investigation and concluded that there is no evidence of misconduct. However, C is under mounting pressure from his dissenters to withdraw the strong claims made in his paper. While C is clear about his honesty in his work and its reporting, because of the strong pushbacks C felt that he might have over-interpreted his data. In discussion with his close colleagues and the journal editor, C eventually initiated a retraction of the paper, citing a loss of confidence in his original interpretations. The retraction notice carries a brief statement on the institution’s investigation results, but the “no misconduct” verdict in the statement was met with doubts and cynical murmurs of sloppiness and cover up, questioning as to why the paper is retracted when there was no RM.

## Deflection of Responsibility

Case 1 depicts a RM-positive case that was duly investigated with the perpetrator identified and convicted. The retraction notice thus carries a statement on the investigation and its outcome. Such is perhaps the proper and ideal scenario that Xu et al. advocates. There might well be an argument on redundancy of information, and on whether the potentially career-terminating public naming and shaming of the young perpetrator would be too harsh. However, given that many institutions do not make their investigations reports public, the retraction notice may well be the only instance in which the research community would have a formal statement on the perpetrator and the latter’s acts that undermined the validity of the paper. Disclosure of institutional investigation would thus be desirable.

A less obvious consequence of the above scenario is that the focus is somewhat unwittingly shifted onto the junior perpetrator of RM, who takes the full blame. The senior corresponding author A is removed from the spotlight and, with refined workings as well as social or community influences, could effectively deflect the responsibility of RM from himself to the perpetrator (for example, by picturing himself simply as a victim of the latter’s deceit). Such a scenario is not uncommon in real life cases, but a case in which the senior person or a principal investigator shirking, or being allowed to shirk responsibility, would be undesirable. While we are not advocating for retaliatory sanctions to be implemented across all contexts, self-reflections on deficits in research quality control and rigour, as well as implementation of changes and improvements to the above, would be the very least expected from the leaderships in research. If a mandate on disclosure of institutional investigation fails to effect the above on the part of the group leaders, principal investigators and senior corresponding authors, the mandate would not have fully served its purpose in promoting transparency.

## Epistemic Injustice

Case 2 illustrated the plight of an author who, in an attempt to correct an honest mistake, found himself instead unwittingly courting an unwarranted RM investigation and a possible retraction. The latter is apparently also not an uncommon situation (Hosseini et al., 2018). There is no way to reverse the decision to open a RM investigation because he has approached the journal, who wanted the institution involved to determine if there was RM. B had simply wished to correct his scientific error and has nothing to hide, but is keenly aware of how an RM investigation could derail his work and career. He was especially worried about how he would face his colleagues who pointed out the error, whom he holds in high regard, as well as others in the field. He knows that during investigation he would not be allowed to discuss case details publically, and the fear of being stigmatized as a cheat even if he is eventually cleared of RM gave him sleepless nights. In this case, the mandate for institutional investigations, if followed through blindly as a requirement, has generated epistemic injustice and confusion that might harm the author.

For case 3, there was an official complaint and as such an institutional investigation is definitely warranted. However, should the RM finding be negative, would the inclusion of a disclosure of institutional investigation in the retraction notice be necessary (for this case the author’s own lack of confidence in his theoretical analyses of his data)? The author had initiated the retraction due to his own loss of confidence in his original interpretation, not due to RM. The RM investigation is therefore irrelevant to the cause of retraction, and it might be better for the investigation findings to be announce elsewhere instead of being bundled with the retraction notice, as this could cause confusion of the facts.

It could be discerned from cases 2 and 3 that a mandatory inclusion of institutional investigation disclosure in retraction notices could result in confusions that causes undue distress to the RM-negative authors and their institutions. Without such a mandate, the retraction notices would have simply stated the proximal intellectual reasons for retraction. There is a certain degree of epistemic injustice (Fricker, 2007)^6^, as suspicions of RM would likely overwhelm the intellectual reason for retraction in terms of public interest and perception. This bias, coupled to gag orders that usually accompany formal investigations, would effectively prevent the parties concerned from engaging in public discussion and debate on the details of their cases. Rumours and hearsays will further influence public opinion. As such, beyond the perceived harm to researchers and their institutions, public trust in science could also be affected if people fail to appreciate or distinguish the actual intellectual issues from allegation or insinuations of RM.

### A More Nuanced Approach– Decoupling or Delaying Institutional Investigation Disclosure From the First Instance of Retraction Announcement

The discussions above illustrate how researchers who made honest mistakes in their work or in reporting research could potentially be harmed by a mandatory disclosure of institutional investigation in the retraction notice, even if such investigations found no evidence of misconduct. One might argue that the above scenarios are circumstantial, and should there be rare instances of epistemic injustice, the harm would be insignificant as the number of retractions due to honest errors are generally perceived to be small compared with those due to RM. However, this number is small only when considered in comparison with RM-positive cases. Fang and colleagues’ analysis of more than two thousand retractions in 2012 has indicated that 67.4% of these could be attributed to RM (Fang et al., 2012), while later analyses with smaller samples in 2016 (Moylan & Kowalczuk, 2016) and 2019 (Campos-Varela & Ruano-Raviña, 2019) returned values of 76% and 65.3%, respectively. In other words, one-third to one-quarter of retractions are due to non-RM-type errors of some kind. Leaving aside the small minority that are retracted because of publishing errors by the journals, we do indeed have a significantly large number of retractions that likely stemmed from honest errors by the authors.

Another obvious issue with mandating a disclosure of institution investigation in retraction notices is that formal institutional investigations would inevitably be time consuming^7^. It has been widely lamented that retractions could take a long time (Bolland et al., 2022; Peng et al., 2022; Torjesen, 2022; Sohn, 2023), and one reason for this would the preference for some journals to wait for formal misconduct investigations to be concluded (Loadsman, 2019). To hasten the retraction process, H. Holden Thorp has recently suggested that investigations should be a two-stage process, with the first stage aiming to simply “… *evaluate the validity of the paper without attributing blame*”. Thereafter, the institutions can delve into more “… *lengthy and more complicated investigation of the underlying wrongdoing*” (Thorp, 2022).

Following Thorp’s cue on the matter, I hereby suggest a similar decoupling of the disclosure of institutional investigation from retraction notices, or a suitable delay in the former’s inclusion. If an institutional investigation has been completed at the point of paper retraction, it should of course be disclosed in the notice. However, if investigations have not yet been conducted or completed, a retraction should require neither such a disclosure, nor be unduly delayed by the lack of one. The notices, at the first instance, need only to state the proximal cause for retraction, which should be a sufficiently detail explanation on (with the key evidence presented) why data/results/conclusions are no longer deemed valid or trustworthy. This would announce the retraction of a paper based on its intellectual and factual demerits, not whether RM has occurred or otherwise. If there is no RM involved, or that no instances of RM is subsequently found in more detailed institutional investigations, the retraction notice should remain as it is on record. However, more elaborate descriptions on findings of RM should be added to the notice retrospectively as an addendum when the institutional investigations are concluded and the reports finalized. The research institution and the journal should ensure that the latter is furnished with at least a summary of the report, which shall enable it to add a sufficiently informative excerpt of the investigation outcome to the original retraction notice, even if the institution has no plans of eventually making the full report public. The addendum shall serve to complete the mandate on institutional investigation disclosure, but such a disclosure needs only to be placed on record if RM is relevant and fully adjudicated as such.

## Concluding Remarks

In lauding the proposed mandate on the disclosure of institution investigation outcome to retraction notices, I have pondered on potential issues and feel that it could be done in a more nuanced manner. The above suggested approach to writing retraction notices would ensure and promote timeliness of retractions (when there is clear evidence for loss of confidence on the factual integrity of the paper) without these being dependent on, or having to wait for, the outcome of lengthy formal investigations on misconduct. Furthermore, the stated proximal cause of retraction in the notice would allow other researchers and the public to be confident of the fact that the self-correcting mechanism of science has been duly activated. An addendum disclosing RM findings from institutional investigations added retrospectively to a retraction notice after investigations are duly completed would provide closure to any suspicion or allegation of RM, and could save authors and institutions of RM-negative cases from distresses and embarrassments caused by epistemic injustice.

## Footnotes

1 Such retractions usually happen as authors realized, retrospectively, that errors have occurred in their analysis (for an example, see one such recent notice in the Journal of Neuroscience (10.1523/JNEUROSCI.0913-22.2022).

2 For an example of an editorial retraction, see the recent retraction in *Science* on Majorana fermion (https://www.science.org/doi/10.1126/science.adf7575), in which the journal has decided to retract the paper despite some authors disagreeing with the decision.

3 Even spontaneous confessions or self-proclamations of misconduct would be investigated and the evidence assessed.

4 This would be in accordance with the COPE guideline of “making a substantial contribution to the work and being accountable for the work and its published form” (https://publicationethics.org/resources/discussion-documents/authorship) and the International Committee of Medical Journal Editors (ICMJE) authorship guideline of “Agreement to be accountable for all aspects of the work in ensuring that questions related to the accuracy or integrity of any part of the work are appropriately investigated and resolved” (https://www.icmje.org/recommendations/browse/roles-and-responsibilities/defining-the-role-of-authors-and-contributors.html#two). The principal investigator or the senior author should take full responsibility as it is with their endorsement that fraudulent contents are included in a paper.

5 Despite the vignettes being fictitious, readers might note that the depicted scenarios resonate with those in the real world.

6 The term “epistemic injustice”, which is perhaps most prominently explained by Miranda Fricker’s seminal book (Fricker, 2007), refers to unfairness related to trusting someone’s word, or that one’s sufferings or grievances are unfairly made less intelligible. It is used here to broadly indicate the injustice in knowledge that could be experienced by researchers that have made honest mistakes but being perceived instead as perpetrators of RM.

7 Jeremy Fox has estimated the time taken for the investigations of 14 famous cases of RM, and reported a range of three months to two years (mean and median time for these is ~ 1 year) in his blogpost (https://dynamicecology.wordpress.com/2021/03/15/how-long-do-institutional-investigations-into-accusations-of-serious-scientific-misconduct-typically-take-heres-some-data/).
